# Convergence or Divergence: Preferences for Establishing an Unemployment Subsidy During the COVID-19 Period by Taxing Across Earnings Redistribution in Urban China

**DOI:** 10.3389/fpsyg.2022.852792

**Published:** 2022-06-10

**Authors:** Yaping Zhou, Jiangjie Zhou, Yinan Li, Donggen Rui

**Affiliations:** ^1^Department of Sociology, Lanzhou University, Lanzhou, China; ^2^School of Management, Lanzhou University, Lanzhou, China; ^3^Institute of Regional Studies, Pukyong National University, Busan, South Korea

**Keywords:** unemployment subsidy, COVID-19, preferences, earnings redistribution, China

## Abstract

With the advancement of marketization, China has achieved rapid economic growth and economic class differentiation. This research analyzes the data from China’s livelihood survey, divides the urban Chinese into five socio-economic classes, and tests their preferences and tendencies for income redistribution. It obtains the general attitude differences in subsidy policy and income inequality during COVID-19. Our conclusion are consistent with the existing literature to a great extent; that is, personal factors (self-interest and belief in fairness) play a crucial role in the attitude of Chinese citizens. In the analysis of situational factors, the results show that the higher the level of marketization, the people are more likely to have stronger negative emotions about subsidy or redistribution policies. Further analysis shows that people with the lowest income are susceptible to the fact that income inequality has become significant and show a strong willingness to support the government’s redistribution policy. In contrast, middle-class people tend to favor the government’s redistribution policy, although they will not benefit much from the redistribution policy. Therefore, they lack the motivation to support the government in vigorously implementing the subsidy policy. Significantly, high-income people are indifferent, as they lack such motivation even more. The difference in redistribution preferences between upper-class and lower-class groups signals polarization in Chinese society, especially income redistribution.

## Introduction

Research on residents’ redistribution preferences has become a hot topic with the continued severity of global income inequality. In recent years, COVID-19 has become a significant public health event that has impacted the global economy and global income inequality. Although research on the redistribution preferences of residents is fruitful, research on the redistribution preferences of residents in the context of COVID-19 is not sufficient. Previous studies have made important discoveries, mainly focusing on the following aspects. First, studies explore residents’ redistribution preferences from the perspective of individual subjective cognition. Studies have shown that the redistribution preferences of Chinese residents are affected by the motivation of economic interests and are significantly affected by the perception of social fairness (Xu and Liu, 2013; Xiao, 2021). Reducing residents’ sense of income fairness enhances redistribution preference and further increases redistribution preferences by reducing the intermediary variable of mobility expectations (Li and Lv, 2019). Some factors influence preferences to redistribute from distributional processes; people have fairness preferences regarding outcomes and how to achieve those outcomes (Tinghög et al., 2017). Second, studies observe residents’ redistribution preferences from the social class perspective (Li Y., 2020). Humanizing high socio-economic status groups leads to lower support for income redistribution for wealthier groups (Sainz et al., 2019). The above studies mainly focus on the individual level, and the redistribution preferences of residents are less explored at the situational level, especially the interaction between the individual and the situational level. Given the impact of COVID-19 and rising regional income inequality, are residents’ redistribution preferences affected by these changes in scenarios? From the perspective of economic stratification, this study explores how urban residents’ redistribution preferences embedded in regional income inequality under the COVID-19 shock change with the context, whether it converges or diverges.

Compared with the impact of the SARS epidemic in 2003, the impact of the 2019 COVID-19 outbreak on employment was more severe and complex, mainly due to the profound changes in the employment pattern. In the service industry, flexible employees with no clear employment relationship, the increasing number of people employed in microenterprises, and migrant workers have become the leading employment groups. However, the employment protection and social security systems are insufficient to cover them. Therefore, Zhang and Wu (2020) estimated that new jobs decreased by 1.4216 million, 4.7792 million, and 6.7861 million, respectively, under the three optimistic, neutral, and pessimistic expectations. In China, the new job losses were 8.70, 29.26, and 41.55%, respectively, affected by COVID-19 in 2020.

By February 2020, infected people had appeared in all provinces of China. As a result, the Chinese government has taken a serious view of the epidemic, while it also stresses the epidemic’s impact on the economy and people’s livelihoods. As a result, the government comprehensively issued many policies to promote epidemic prevention and control and economic development. Among these, many practices are worthy of in-depth understanding and research.

For instance, in response to the abovementioned relative reduction in employment, the government has established an unemployment subsidy policy. Traditionally, the evaluation of the effect of a public policy tends to evaluate its efficiency, and citizen response or citizen attitude will not be the most important in the evaluation. However, in China, with such a large population base, the authors believe that the attitude of citizens toward such a policy that benefits the people’s livelihood but does not involve all citizens is still of great research value. What factors will affect the presentation of their relevant attitudes? Will their attitudes change over time? These problems deserve the attention and discussion of scholars and policymakers, and what is more, they need to respond to them.

Using data from the China People’s Livelihood Survey, this research, from the perspective of socio-economic class, attempts to explore how the two factors affect urban residents’ attitudes, inclinations, and preferences for unemployment subsidies. Individual-level factors include self-interest and belief in fairness, and contextual factors include time and region. In addition, this research aims to explore the employment redistribution preferences of Chinese urban residents of different economic classes.

This study attempts to make a small contribution from three aspects. First, theoretically, the perspective of economic stratification is introduced based on integrating the previous perspectives of self-interest and fairness belief analysis. As income inequality continues to increase during China’s transition period, it is more appropriate to explore whether to establish unemployment benefits from the perspective of economic stratification during the new crown epidemic. Second, in terms of research methods, contextual influencing factors, based on presenting individual influencing factors, such as the Gini coefficient and marketization index, are included in the analysis variables. Then we further conduct interaction analysis about individual and contextual factors. Third, in terms of policy, data analysis in 2020 and 2021 outlines the changes in people’s attitudes toward tax collection and unemployment benefits in different economic classes. The spread of COVID-19 provides policy intervention for the government direction. At the same time, we also need to determine whether the Chinese society has formed a consensus attitude toward unemployment benefits and whether there is any danger of polarization or even rupture.

The structure of this research is as follows: The first part is a brief introduction, and then we describe the origin of the research and puts forward hypotheses through background and literature review. The fourth part explains the source and processing method of the data, and the fifth part clarifies the analysis results. Finally, we discuss the result in detail in the sixth part, and the last part provides a summary and limitations.

## Background of the Study

According to the output loss conversion method, Gao (2020) estimates that COVID-19 will bring approximately 23 million job losses in 2020. As a result, total employment decreased by approximately 7.5 million compared with the previous year, equivalent to increasing the unemployment rate by approximately one percentage point. Furthermore, accommodations, catering, tourism, entertainment, and transportation lost millions of job opportunities in the second and third industries.

The outbreak of COVID-19 has also significantly impacted the employment of new graduates of colleges and universities. Comparing the psychological pressure and employment choices of new graduates before and after the outbreak, scholars found that the epidemic has negatively affected new graduates in many aspects. For example, they met recruitment interview obstruction, job implementation decline, high employment pressure, and pessimistic expectations for the future economy (Li C., 2020). The COVID-19 epidemic has had a significant impact on the employment of college graduates. This impact is functional and structural, not only in the short term but also in the long term. The epidemic’s impact not only exists in the group of new graduates, which has brought new challenges to employees’ adaptation to changes and job security and has dramatically affected their wellbeing and satisfaction. The survey and empirical data analysis of 568 employees in Romania show that there is a negative correlation between job instability and job satisfaction (Nemteanu et al., 2021).

The global outbreak of COVID-19 has deeply damaged the industrial chain, supply chain, and financial chain. All the countries have continuously introduced various policies to restore economic development. Mainly focused on increasing the government’s fiscal deficit and debt has attracted much attention. From the views of modern monetary theory and functional finance, government deficit spending reflects the essence of functional finance.

Reducing market interest rates has the effect of crowding in investment, whereas it stabilizes prices and promotes full employment. However, liberal economists and political thinkers have attacked the idea of public good for a long time. New scientific models and post-structuralist ideas from the start of the 20th century helped people reconnect with the idea of the public good to guide politics, accommodate liberty and diversity, and overcome liberal objections (Olssen, 2021). Therefore, the sovereign government can and must actively act as the last resort and implement the employment guarantee scheme to ensure employment and stable growth.

The Chinese government wants to improve the effectiveness of the employment stabilization measures and effectively deal with the epidemic’s impact. It has also realized that we should focus more on vulnerable groups during the policy implementation and create a relief model to achieve good governance.

China has faced a problematic employment situation in recent years, especially since the outbreak of COVID-19. Social insurance premiums have inherent defects, making it challenging to meet the future development needs of China’s employment situation. Some researchers have pointed out that social insurance should reform to meet the needs of China’s employment situation development through “fee-to-tax” (Wu and Zhou, 2020).

Since the outbreak of COVID-19, enterprises in China have faced the problems of extended downtime and difficult resumption of work. As a result, in February 2020, the national urban survey unemployment rate peaked at 6.2%, an increase of 0.9% over the same period in 2019. In addition, the number of new college graduates in 2020 reached an all-time high of 8.74 million (China Ministry of Education, 2019; China National Bureau of Statistics, 2020). There is no doubt that China’s employment situation is bleak against the background of the epidemic. Therefore, the Chinese government has issued various policies and measures to stabilize employment, such as issuing unemployment subsidies during the epidemic to alleviate employment difficulties. The unemployment subsidy policy is a particular policy issued in a unique period. Its primary purpose is to increase the living assistance to the unemployed under particular circumstances and ensure their basic life.

In February 2020, the Ministry of Human Resources and Social Security, the Ministry of Finance, and five other departments issued a notice on doing an excellent job in employment during the epidemic prevention and control period. Furthermore, making it clear that areas with serious epidemic situations, such as Hubei, grant unemployment subsidies to insured unemployed persons who do not meet the statutory conditions for receiving unemployment insurance benefits. In March 2020, the general office of the State Council issued the implementation opinions on strengthening measures to stabilize employment in response to the epidemic’s impact. They made it clear that in 2020, if an unemployed person did not meet the statutory conditions for receiving unemployment insurance benefits, while the unemployment insurance is about to expire, they were granted unemployment subsidies for 6 months. The standard for the subsidy is not higher than 80% of the local unemployment insurance benefits. The implementation of this new policy has expanded the current scope of unemployment protection to all insured unemployed persons, from areas with severe epidemics to the whole country.

During the COVID-19 period, the government issued more unemployment benefits for more unemployed people, which has become a vital welfare measure in many other countries. For instance, in America, in March 2020, President Trump signed the Families First Coronavirus Response Act (FFCRA), which provided additional administrative funds for the response to the COVID-19 epidemic. Expanding unemployment insurance during the economic downturn is a standard policy practice in the United States, not only in the COVID-19 crisis but also in the Great Recession. This law is similar to China’s unemployment insurance policy mentioned above. Both try to enhance their resilience by providing unemployment insurance for workers who are not entitled to unemployment benefits to cope with the unemployment pressure brought by COVID-19.

During the shutdown period, the Spanish government also implemented the policies of unemployment benefits and social bonuses for electricity to help people alleviate energy poverty. According to the research results of several cases during the shutdown period in Spain, the increase in unemployment benefits should be especially applicable to the shutdown period with unemployment, difficulty in finding a job, increase in residence time, and increase in energy consumption and expenses caused by the epidemic (Bienvenido-Huertas, 2021).

According to the public opinion survey on the German unemployment insurance system, the researchers believe that it is appropriate to increase income through unemployment insurance before and during the COVID-19 crisis. A specific insurance period can stabilize the income status of welfare recipients and provide time to find appropriate jobs to improve the quality of suitable employment (Osiander et al., 2021).

The unemployment subsidy policies have achieved specific results; however, what is the public’s attitude toward this new redistribution policy? There are a few empirical studies on people’s attitudes toward the epidemic subsidy policy, especially in China. From experience, benefitting from the unemployment insurance system established in 1986 and lasting for many years, China has a public opinion basis for extending unemployment benefits. However, China has always had an egalitarian tradition of “not suffering from oligopoly but inequality.” Moreover, that tradition has almost become a collective subconscious social development attitude. Equalitarianism has become the background of populism, which negates the rational development of diversification by pursuing the equality of interests. This populist tendency especially embodies the people’s demands at the bottom. Populists among the bottom classes are even more (Xu, 2018). As a result, the differentiation of economic classes may lead to different attitudes toward unemployment subsidy policies among different economic classes.

In terms of examining factors influencing Chinese urbanites’ attitudes toward COVID-19 subsidies, their preferences for redistribution, one of the public opinions, can be clearly explained by subsiding themselves, representing a type of income redistribution. Specifically, address the issue of class polarization in urban China. In other words, how do Chinese citizens of different social classes have different preferences for redistribution? Furthermore, regarding critical values, such as redistributive needs, does the attitude of Chinese citizens become more decentralized or centralized? Finally, we should consider class differentiation’s degree, nature, and trajectory.

## Literature Review

At present, Chinese society has entered a severe period of income inequality, with populist tendencies impacting employment funds. To this end, the Chinese government has reformed the national tax system to alleviate the income inequality gap.

The researchers examined China’s two tax reforms from progressive and social welfare. They found that the abolition of agricultural tax reduction significantly improved the social welfare of rural residents. Although the income tax threshold increases the progressive rate, it reduces the overall share of income tax in the total tax. Therefore, both reforms have raised the overall welfare level. The results show that due to its decreasing nature, agricultural taxes inhibit the welfare of low-income groups and increase inequality. Therefore, the cancelation of agricultural taxes positively impacts farmers’ income gap between urban and rural areas. Although the income tax is progressive, its share in the total tax is tiny, reducing its redistribution effect. After the tax reform in 2011, with the increase in the income tax threshold, the positive role of redistribution was further weakened. However, because it exempts many low-income groups from income tax and the marginal value of income of the poor is much higher, the overall social welfare has increased. The researchers predict that the tax reform implemented in early 2019 (introduced in 2018) will further reduce the tax burden on middle-income people (Shen et al., 2021).

Using the microsimulation model of China’s income tax (PIT), the researchers compared the personal income tax systems in 2011 and 2018. They found that residents from different sources of income may face a significant degree of changes in the effective tax rate. Once the tax system changes to PIT 2018, the income redistribution effect will reduce from 1.95 to 1.22%. The role of PIT in fiscal revenue will also be negatively affected, and its income redistribution function will be challenging to recover in the short term. However, the researchers also found that the impact of PIT on income distribution depends on the tax structure. Under appropriate conditions, the gradual transition to a “comprehensive” tax system will achieve a better income redistribution effect with a lower average tax rate. However, from the perspective of social equity, a higher exemption rate does not necessarily create an equal system. Excessive tax exemption will reduce the tax burden of some groups, but it will also significantly reduce the income redistribution function of PIT. In that regard, we should avoid excessive emphasis on the role of immunity (Zhan et al., 2019).

With a basic understanding of China’s tax policy, we further discuss the attitude toward tax policy. There have been many studies on the attitude of tax policy in academic circles. Previous studies have documented that Americans tend to be victims of “enlightened self-interest” when evaluating complex national tax policies. However, recent studies have shown that the simplicity and clarity of emerging local redistribution initiatives promote the formulation of economic self-interest, especially among lower-income citizens. Consequently, low-income citizens have the highest support for their progressive tax policies (Newman and Teten, 2021).

Both self-interest motivation and political ideology are essential factors to explain the preference for tax plans. Through the multilevel model to test socio-economic status and political ideology to estimate personal preference, the financial burden affects the preference for tax plans (straightforward tax) and interacts with self-interest and ideological variables. Under a higher level of direct taxation, it is possible to support the redistribution of the poor and the rich. With political factions becoming highly polarized, left-wing political parties have strengthened their ability to mobilize voters further to pursue their redistribution interests. In contrast, right-wing voters have increased their resistance to taxing the rich (Jaime-Castillo and Sáez-Lozano, 2016). Similarly, in the study of support for the tax reduction and Employment Act, a similar conclusion was also reported; self-interest and partisan prejudice will play a role, but partisan relations are even more critical (Mendoza Aviña and Blais, 2022).

In the context of Washington state’s proposal 1098, a study shows how economic self-interest, concerns about inequality, and partisan bias affect support for redistributive taxes. The results report that all these factors affect the support rate. Nevertheless, the support rate of low-income people is very high, which shows that when the distribution policy meaning is clear, citizens can transform their interests and general attitudes into consistent redistribution preferences (Franko et al., 2013).

There are a few studies on this issue in the context of China. However, there are many cases of the attitude of unemployment tax or unemployment subsidy policy during the epidemic in the literature of South Korea.

A study verified the policy feedback theory that individual social policy experience affects welfare attitude. The relationship between disaster relief fund satisfaction and welfare attitude confirmed that disaster relief fund satisfaction and policy effectiveness affect welfare attitude in the overall and male models. However, the study found no regulatory effect on policy effectiveness. In the female model, satisfaction with disaster relief funds and policy effectiveness affect welfare attitude, and policy effectiveness has a negative regulatory effect (Moon and Sun, 2021).

In the comparative study on the comments of South Korean and Japanese citizens on the COVID-19 emergency relief fund, the researchers found that South Korean and Japanese citizens are generally sensitive to the tax problems of foreigners. However, if immigrants fulfill their obligations, they can obtain state welfare and support, similar to indigenous people (Rin, 2021). Another study on the citizens of Daegu, South Korea, shows that providing disaster relief funds can improve the region’s sense of belonging and pride to stimulate communication among residents and jointly overcome difficulties (Kim et al., 2020).

From the relevant literature thus far, we found that to probe this issue, we need to consider both individual- and situational-level factors concurrently while exploring the reasons for people’s preference redistribution. Therefore, from the discussions above, we issue the following hypotheses.

### Individual Factors-Based Hypotheses

H1. *Compared with the current low-income group, the current high-income group–as measured by current personal incomes and current comparison incomes with others–is more likely to be less supportive of the employment subsidy policy during the COVID-19 period.*

H2. *People with higher income expectations or those with lower income in the past are more reluctant to support the establishment of employment subsidies during the COVID-19 period than those with lower future income prospects or higher evaluation in the past.*

H3. *Compared with those who have experienced downward mobility in the past or have lower expectations of upward mobility in the future, those who have experienced social mobility upward or higher expectations of upward mobility have a lower tendency to support the employment subsidy policy during the COVID-19 period.*

H4. *The more people emphasize the importance of diligence and effort to success and wealth, the lower the level of support for establishing employment subsidies during the COVID-19 period.*

### Situational Factors-Based Hypotheses

H5. *The higher the level of objective income inequality (Gini index), the more people approve of the unemployment subsidy policy during the COVID-19 period.*

H6. *The higher the degree of marketization, the more reluctant people are to support the unemployment subsidy policy during the COVID-19 period.*

H7. *The more significant the development of income inequality (Gini index), the more people endorse the unemployment subsidy policy during the COVID-19 period.*

H8. *The more prosperous the development trend of the marketization level, the more people tend to agree with employment support during the COVID-19 period.*

## Data and Method

### Dataset and Samples

The following analysis develops from the 2020 and 2021 social surveys launched by Huazhong Agricultural University and Lanzhou University. Eight Chinese universities jointly conducted a nationally representative survey. The survey adopted the PPS sampling method, and the respondents were 18 years and older in China. There were 4,694 cases in the study sample in 2020 and 5,205 cases in 2021. The quartile division method divides the people participating in the survey into five levels, namely, the top, middle-top, middle, middle-bottom, and bottom. The variables involved in the study are listed in the Supplementary Tables for better clarification.

### Method

This study used two primary statistical approaches, including a description of polarization and a generalized linear model.

As far as the first approach is concerned, given the significance of distribution properties of public opinion in the study of politics and subgroup relations (Di Maggio et al., 1996), a multidimensional definition of attitude polarization is developed. Di Maggio et al. (1996) summarized four aspects of opinion aggregation as a foundation for measurement, and we use two of them.

1. The dispersion principle: “Other things being equal, the more dispersed opinion becomes, the more difficult it will be for the political system to establish and maintain centrist political consensus” (Di Maggio et al., 1996, p. 693);
2. The bimodality principle: “Other things being equal, the greater the extent to which opinions move toward separate modes (and the more separate those modes become), the more likely social conflict will ensue” (Di Maggio et al., 1996, p. 693).

Accordingly, we used two specific indicators to measure dispersion and bimodality.

First, “polarization” describes how public opinion on an issue is diverse, deviant, and balanced between two extremes of the opinion spectrum. The core of the dispersion is variance, which measures the difference in opinions of any two people influenced by extreme cases (Di Maggio et al., 1996). The formula for variance is


$$S^{2}=\sum X-X^{2}/\left(N-1\right)$$

Second, “bimodality” is used to delineate polarized public opinion, which refers to the situation in which people hold different opinions toward an issue, cluster into separate groups, and locate themselves between the two extreme positions sparsely occupied (Di Maggio et al., 1996). It should be clear that bimodality is different from the distance between positions since “the extent to which opinion variation leads to conflict is likely to depend on the extent to which occupants of polar stances are isolated from one another” (Di Maggio et al., 1996, p. 694). The formula for bimodality is


$$K=\frac{\left[\sum \frac{X-m^{4}}{N}\right]}{S^{4}}-3$$

where *m* is the mean, *s* represents the standard deviation, and subtracting “3” guarantees that the normal distribution takes the value “0.”

Specifically, “variance” stands for the spread of opinion, while “kurtosis” represents bimodality (Walker and Lev, 1969, chap. 4). The former, calculated from the average squared difference of each value from the mean value, is a measure of dispersion and polarization (United Kingdom, SPSS; Di Maggio et al., 1996). “Kurtosis,” the latter indicator, is sensitive to extreme values and can distinguish between a sharp skew to either side and movement of values from the center to both ends of the distribution (Di Maggio et al., 1996). Another indicator, “skewness,” in terms of the direction in which stretched out the tails of the peak (or peaks), can indicate the direction in which a distribution deviates from normality (United Kingdom, SPSS; Di Maggio et al., 1996).

The second approach in this study is the hierarchical generalized linear model (HGLM), which simultaneously estimates individual- and situational-level effects. The data are first hierarchically organized with individuals in terms of five economic class groups nested within provinces and with selected information at both the individual and situational levels. Then, we used the data to estimate people’s attitudes in urban China toward establishing employment subsidies during the COVID-19 period. The dependent variable (i.e., attitude toward establishing employment subsidies during the COVID-19 period) is dichotomous, with two outcome values, 0 or 1.

The specification at the individual level is:


$$log\left[j_{\mathrm{jj}}/\left(1-j_{\mathrm{jj}}\right)\right]=b_{0j}+b_{1j}\mathrm{control}\mathrm{variable}+b_{2j}\mathrm{self}\mathrm{interest}\mathrm{belief}$$

where *j*_*ij*_ is the probability that respondent *i* in province *j* supports establishing employment subsidies during the COVID-19 period, and *b_1*j*_, b_2*j*_*, and *b_3*j*_* are the coefficients for indicators of control variables, self-interest, and fairness belief, respectively.

The specification at the situational level is:


$$b_{0j}=g_{00}+g_{01}\mathrm{marketization}\mathrm{index}\mathrm{Lev}2+g_{02}\mathrm{Gini}\mathrm{coefficient}\mathrm{Lev}2+u_{0j},u_{0j}∼N\left(0,t_{00}\right)$$

where *g*_00_ is the average logarithmic odds of establishing employment subsidies during the COVID-19 period across provinces and *t*_00_ is the variance between provinces in the average logarithmic odds of supporting establishing employment subsidies during the COVID-19 period. All non-dummy individual-level independent variables are grand-mean centered, creating a variable with a mean of zero across all cases.

Given the lack of a Gini coefficient for each province in China, we cannot completely achieve the situational level’s hierarchical generalized linear model (HGLM). Thus, its formula is modified as follows:


$$b_{0j}=g_{00}+g_{01}\mathrm{marketization}\mathrm{index}\mathrm{Lev}2+u_{0j},u_{0j}∼N\left(0,t_{00}\right)$$

To overcome this insufficiency, we estimate the Gini coefficient of each province based on the individual earnings of the sample and their changes over time.

## Results

**FIGURE 1:**
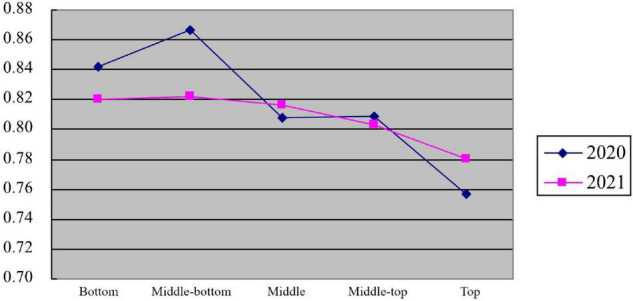
Percentage of five economic classes supporting establishing unemployment subsidy during the COVID-19 period in 2020 and 2021.

Figure 1 shows the proportion of urban residents in China who expressed support for the government imposing unemployment subsidies during the COVID-19 impact period (2020–2021). Axis *X* represents five different economic classes, and axis *Y* represents the proportion of respondents who support the unemployment subsidy policy in the total number of respondents in their economic class.

Overall, the proportion of people who support the government establishing employment subsidies over the 2 years correlates with the respondent’s socio-economic class. There were significant differences in respondents who supported government employment subsidies across socio-economic classes from 2020 to 2021. Respondents at the middle and upper economic levels show the most significant change in attitude over the 2 years.

Table 1 presents the fundamental statistical indicators of respondents’ attitudes toward unemployment benefits in the 2 years affected by COVID-19. In 2020 and 2021, the mean values are 0.8165 and 0.8088, respectively, with corresponding variances of 0.150 and 0.155. A succinct explanation is that in the first year of exposure to COVID-19, most respondents’ attitudes do not differ much, while they show a more significant divergence after 1 year.

**TABLE 1:** Polarization characteristics for 2020 and 2021.

Year	2020	2021
Mean	0.8165	0.8088
Variance	0.150	0.155
Skewness	−1.636	−1.571
Std. error of skewness	0.036	0.034
Kurtosis	0.676	0.469
Std. error of kurtosis	0.071	0.068
*N*	4694	5205

In these 2 years, the proportion of respondents with a positive kurtosis (2020: 0.676; 2021: 0.469) suggests that the attitudes of Chinese urban residents toward the government establishing unemployment subsidies are broadly consistent, and the public reaches a consensus. At the same time, the data skewness tends to be lower in both years (2020: −1.636; 2021: −1.571), showing that more people start to express their positive attitude toward unemployment subsidies in 2021.

Table 2 uses hierarchical generalized linear modeling (HGLM) to estimate Chinese urban residents’ attitudes toward the government’s unemployment subsidy policy in 2020. Model 1 includes two factors: the respondents’ socio-economic class and the level of marketization. The socio-economic class significantly impacts respondents’ attitudes, with most urban Chinese in the higher classes having negative attitudes toward unemployment subsidies during COVID-19. In contrast, middle-bottom class people are almost two times as likely to support the government establishing unemployment subsidies (2.159). Additionally, included in Model 1 is the marketization index, which is a measure of the context in which the respondent lives. After controlling the other independent variables, the marketization index significantly correlates with the proportion of Chinese urban residents who support unemployment subsidies during the COVID-19 epidemic. For example, for every one-unit increase in the marketization index, respondents are 1.7% less likely to support the establishment of unemployment subsidies. Overall, Model 1 shows that the average probability of all Chinese urban residents supporting COVID-19 unemployment subsidies is 0.36.

**TABLE 2:** Hierarchical logistic regressions coefficient predicting odds of supporting establishing unemployment subsidy during the COVID-19 period on individual-level and situational-level variables in 2020.

	Model 1		Model 2		Model 3		Model 4	
	B	Odds	B	Odds	B	Odds	B	Odds
***Economic class* (reference = top)**								
Bottom	0.601***	1.824	0.596***	1.815	−0.370*	0.691	−0.501	0.606
Middle-bottom	0.770***	2.159	0.631***	1.880	0.122*	1.130	−0.063	0.939
Middle	0.316**	1.372	0.174	1.190	−0.167	0.846	−0.417	0.659
Middle-top	0.333**	1.395	0.249*	1.282	−0.031*	0.969	−0.150	0.860
***Control variable***								
Age			0.023***	1.023	0.024***	1.024	0.024***	1.024
Gender (reference = male)			0.036	1.037	0.122	0.246	0.147	1.158
Employment status (reference = employed)			−0.123	0.884	−0.226	0.097	−0.129	0.879
Marital status (reference = married)			0.001	1.001	−0.111	0.895	−0.134	0.875
Schooling			−0.043***	0.958	−0.051*	0.951	−0.052	0.950
Welfare			0.004	1.004	0.016	1.016	0.009	1.009
***Individual-level***								
**Self-interest**								
Current personal income (log)					−0.309*	0.735	−0.330*	0.719
**Past comparison income (reference = bad)**								
*better*					−0.234	0.791	−0.228	0.796
*same*					−0.175	0.840	−0.187	0.830
**Current comparison income (reference = bad)**								
*better*					−0.212	0.809	−0.169	0.845
*same*					−0.095	0.909	−0.063	0.939
Intragenerational mobility (edu.)					0.003	1.003	0.010	1.010
**Fairness belief**								
**Existence of the rich and the poor (reference = disagree)**								
*agree*							−0.428***	0.652
**Children’s equal access to education (reference = disagree)**								
*agree*							0.296*	1.344
**Equal opportunities of descents of workers or peasants to become high-socio-economic-status people (reference = disagree)**								
*agree*							−0.307*	0.736
***Situational-level***								
Marketization index	−0.017***	0.983	−0.016***	0.984	−0.017***	0.983	−0.015***	0.985
Gini coefficient	0.039*	0.875	0.036*	0.816	0.028*	0.789	0.022*	0.746
Constant	1.705***		1.214***		4.690***		5.146**	
***Province level effects u_0_***	0.36		0.32		0.28		0.25	
***Chi-square***	5272		5098		4987		4256	
***N***	4694		4650		2510		2172	

***p < 0.001, **p < 0.01, *p < 0.05.

Model 2 includes the two variables from Model 1 and other independent variables, such as respondents’ personal information. After controlling for age, gender, employment, marriage, schooling, and welfare level, the correlation between respondents’ socio-economic class and their attitudes toward COVID-19 unemployment remained the same as in Model 1. Those in the lower-middle socio-economic class are less likely to show a supportive attitude toward the government’s establishment of the COVID-19 unemployment subsidy than respondents in the upper class (1.880). For each new variable, respondents’ age was positively correlated with their supportive attitudes toward the COVID-19 unemployment subsidy (1.023), while schooling was negatively correlated (0.958). The other variables, including gender, employment, marriage, and welfare level, were not significantly correlated with respondents’ attitudes toward establishing subsidies. In Model 2, when other variables are controlled, there is a significant negative correlation between the marketization index and the level of support for COVID-19 unemployment subsidies among urban Chinese residents. In Model 2, the average predicted probability of respondents’ support for the government establishing unemployment subsidies across provinces is 0.32.

Model 3 builds on Model 2 by including factors of the self-interest of respondents, such as their current personal income, relative income level, and education level based on past mobility experiences. Respondents from the middle-bottom class remain the most likely to support the COVID-19 unemployment subsidy policy. In contrast, the attitudes of their counterparts in the middle class toward the subsidy are not significantly correlating with socio-economic class. Among the control variables, age is positively correlated, and education negatively correlates with respondents’ attitudes toward establishing the COVID-19 unemployment subsidy, while the other control variables were not significantly correlated. Among the self-interest variables, respondents’ current personal income negatively correlated with their attitudes toward establishing the COVID-19 unemployment subsidy (73.5%). However, relative income and education levels based on mobility experience did not correlate significantly. The marketization index and people’s attitudes toward the subsidy policy by the government still showed a negative correlation (98.3%). The average predicted probability that respondents in each province would support the subsidy is 0.28.

In Model 4, the variable of beliefs about fairness was added to the model and compared to Model 3. When this individual-level influence is added to the model, the effect of socio-economic class on respondents’ attitudes toward subsidies is no longer significant. Among the control variables, age significantly affected respondents’ attitudes toward subsidies. As age increases, the level of support for establishing the COVID-19 unemployment subsidy increases (1.024), the same as the previous model. In addition, personal income negatively relates to the level of support for unemployment subsidies among urban Chinese. Those with higher wages have a lower probability of supporting the government in setting up COVID-19 unemployment subsidies (0.739). None of the respondents’ self-interest variables significantly affected the results. On the equity belief dimension, respondents were less likely to support the introduction of subsidies if they agreed that the gap between rich and poor was justified (0.652). If respondents believe that their children’s educational conditions are fair, they are more likely to support a subsidy policy than those who believe their children’s educational conditions are not fair (1.344). Respondents who believe that the offspring of workers and farmers have the opportunity to move up the social ladder are less likely to support COVID-19 unemployment subsidies (0.736). Finally, the direction of influence of the MMI on urban Chinese regarding COVID-19 unemployment subsidies remains unchanged. Overall, the average predicted probability of support for subsidies among respondents in all provinces is 0.25.

Table 3 presents the results of the HGLM applied to the 2021 data. Corresponding to Table 2, Model 1 presents the effects of socio-economic class and level of marketization on respondents’ attitudes. People of the middle-bottom level are the most likely to support the COVID-19 unemployment subsidy. In contrast, those of the highest level are the least likely to support the unemployment subsidy.

**TABLE 3:** Hierarchical logistic regressions coefficient predicting odds of supporting establishing unemployment subsidy on individual-level and situational-level variables in 2021.

	Model 1		Model 2		Model 3		Model 4	
	B	Odds	B	Odds	B	Odds	B	Odds
***Economic class* (reference = top)**								
Bottom	0.250*	1.284	0.164*	1.178	0.300*	1.350	0.208	1.231
Middle-bottom	0.266*	1.304	0.234*	1.264	0.259*	1.296	0.184	1.201
Middle	0.227*	1.255	0.172	1.188	0.263	1.301	0.185	1.203
Middle-top	0.137	1.146	0.089*	1.093	0.136*	1.145	0.012	1.012
***Control variable***								
Age			0.004***	1.004	0.002***	1.002	0.002***	1.002
Gender (reference = female)			−0.147*	0.863	−0.153	0.852	−0.202	0.814
Employment (reference = unemployed)			−0.079	0.619	−0.052	0.752	−0.062	0.786
Married (reference = unmarried)			0.031	1.032	0.029	1.021	0.35	1.128
Education			−0.003***	0.997	−0.015*	0.985	−0.013*	0.987
Welfare			0.034	1.035	0.055*	1.057	0.052	1.047
***Individual-level***								
**Self-interest**								
Current personal income (log)					−0.428***	0.652	−0.376***	0.876
**Past comparison income (reference = bad)**								
*better*					−0.187	0.829	−0.197	0.798
*same*					−0.176	0.876	−0.165	0.897
Past promotion experience (reference = Yes)					0.072	1.717	0.065	1.643
Past wage increase experience (reference = Yes)					−0.087***	0.916	−0.076***	0.998
Future promotion expectation (reference = Yes)					0.876	1.983	0.879	1.965
Future wage increase expectation (reference = Yes)					−0.012***	0.876	−0.16***	0.921
Intragenerational mobility (occupation)					0.087	1.287	0.092	1.876
Intragenerational mobility (edu.)					0.098	1.876	0.062	1.246
**Fairness belief**								
Success due to luck							0.098	1.112
Success due to social network							0.092*	1.009
Success due to individual capacity							−0.096*	1.765
**Being poor due to idleness (reference = disagree)**								
*agree*							−0.076***	0.982
**Being poor due to education insufficiency (reference = disagree)**								
*agree*							−0.089	0.971
**Being poor due to the government’s policy (reference = disagree)**								
*agree*							0.125***	1.652
***Situational-level***								
Marketization index	−0.021***	0.972	−0.020***	0.976	−0.019***	0.971	−0.018***	0.981
Gini coefficient	0.032*	0.765	0.029*	0.762	0.022*	0.675	0.019*	0.629
***Constant***	1.268***		1.208***		1.118***		1.109***	
***Province level effects u_0_***	0.37		0.35		0.29		0.26	
***Chi-square***	6785		5878		2987		2675	
***N***	5205		4984		4321		3987	

*P ≤ 0.05, **P ≤ 0.01, and ***P ≤ 0.001.

Model 2 also adds several control variables, which introduces some changes. Those in the middle of the socio-economic class are not related to the attitude toward unemployment subsidies. Three control variables, including age, gender, and schooling, significantly affect Chinese urban residents’ attitudes toward the COVID-19 unemployment subsidy. Specifically, females, those with higher levels of education and those who were older were more likely to support the public policy to establish the COVID-19 unemployment subsidy. The other three control variables, employment, marriage, and welfare level, were not significantly related to respondents’ attitudes toward unemployment subsidies.

In Model 3, we added a new set of variables about self-interest. Analysis shows that Chinese urban residents of different socio-economic levels’ preferences for COVID-19 unemployment subsidies are significantly related to age, schooling, and welfare. Older people are more likely to support the subsidy. Similarly, those with lower levels of education and those with higher levels of social welfare are more supportive of the government establishing the COVID-19 unemployment subsidy. The newly added variables about self-interest are not in the 2020 data, such as respondents’ mobility experiences during the COVID-19 period and their expectations of future mobility status. Specifically, respondents’ previous experience with wage increases and expectations of future wage increases negatively affect their support for unemployment subsidies. However, respondents’ previous experience with promotions and expectations of future promotions do not show significant effects. In Model 2, current personal income significantly affects respondents’ preference for unemployment subsidies (0.652).

The last model in Table 3 incorporates variables on equity beliefs. In this model, the correlation between people’s socio-economic class and subsidy preferences is insignificant. Older, less-educated people were more likely to support the government’s establishment of COVID-19 unemployment subsidies. Respondents’ current personal income, past wage growth experiences, and future wage growth expectations negatively correlate with their subsidy preferences. We operationalize the Equity beliefs into six variables, three of which, luck, social networks, and personal capabilities, were used to measure success. We also use idleness, educational deprivation, and policy support to measure poverty. The results showed that if respondents attribute success to luck or poverty to educational deprivation, their equity beliefs do not significantly affect unemployment benefit preferences. On the other hand, respondents are more likely to support the COVID-19 unemployment subsidies if they attribute success to social networks or poverty to policy failures. Furthermore, they are more likely not to support unemployment subsidies if they believe that success stems from personal effort or poverty stems from the refusal to work (choosing to be idle).

According to the four models in Table 3, the marketability index negatively correlates with urban Chinese preferences for unemployment subsidies (0.972 in Model 1, 0.976 in Model 2, 0.971 in Model 3, and 0.981 in Model 4). The average predicted probability of support for subsidies by respondents in each province is 0.37 in Model 1, 0.35 in Model 2, 0.29 in Model 3, and 0.26 in Model 4.

In Table 4, we report our multilevel model and the estimates. However, we must first clarify that the variables included in this table exist in the 2020 and 2021 surveys since the same questions allow us to combine these two datasets.

**TABLE 4:** Estimated parameters of the preferred multilevel model of attitudes establishing an unemployment subsidy during the COVID-19 period.

	B
***Baseline coefficient***	
Intercept	1.276***
***Economic class* (reference = top)**	
Bottom	0.218***
Middle-bottom	0.277***
Middle	0.102*
Middle-top	0.105*
Age	0.006***
Gender (reference = female)	0.076
Employment (reference = unemployed)	0.065
Married (reference = unmarried)	0.068
Education	−0.011*
Welfare	0.032
Income (log)	−0.398***
***Trend coefficient***	
Wave (1 = 2021)	0.017
***Economic class* (reference = top)**	
Bottom × wave	−0.008**
Middle-bottom × wave	−0.007**
Middle × wave	0.002*
Middle-top × wave	0.005*
Age × wave	0.002***
Gender (reference = female) × wave	0.006
Employment (reference = unemployed) × wave	−0.002
Married (reference = unmarried) × wave	−0.021
Education × wave	0.007*
Welfare × wave	−0.017
Income (log) × wave	0.003***
***Micro macro interactive coefficient***	
Wave (1 = 2021)	0.0267***
***Micro-level variance component***	
Var	0.146

*P ≤ 0.05, **P ≤ 0.01, and ***P ≤ 0.001.

Situational-level variation in people’s preferences for subsidies correlates with age, schooling, and income-level changes. Specifically, the coefficient of schooling is −0.011, indicating a small and negative association of the trend in the level of people’s demand for subsidies with the growth of marketization. The negative coefficient of income reveals that the rise in income is decided mainly by marketization between 2020 and 2021. After controlling for the growth of marketization, the extent to which age accounts for people’s subsidy attitudes is slightly lower in 2021 than in 2020. Among the trend coefficients, the proportion of people from the bottom-level economic class who support subsidies decreases from 2020 to 2021 (−0.008), while that of the middle-bottom similarly drops during these 2 years (−0.007).

In contrast, the proportion of those from the middle-top and top who favor subsidy increases in 2021 (0.005 and 0002, respectively). The only insignificant group is the middle-level economic class. For the control variables, the changes in gender, employment status, marital status, and welfare index during the 3 years from 2020 to 2021 have nothing to do with people’s preferences for subsidies.

In conclusion, we analyzed and demonstrated substantial regional variation in levels of people’s preferences for subsidies, temporal changes in levels of people’s preferences for subsidies, and temporal changes in return to economic class. In particular, this table implicitly shows that for those with a higher level of economic class (i.e., top- and middle-top), there is a positive association between their demand for subsidy and their level of economic class. At the same time, for their lower counterparts (i.e., bottom- and middle-bottom), a negative correlation exists between their subsidy attitudes and the level of their economic class. Finally, in Part 6, we discuss our findings’ theoretical and practical implications.

## Findings and Discussion

As shown in Figure 1, comparing the data from 2020 and 2021, the variance in attitudes toward COVID-19 unemployment subsidies among Chinese urban residents in different socio-economic classes diminishes. This result is because unemployment subsidies are essentially income redistribution measures that the government may take to reduce the negative impact of such an epidemic. Therefore, taking kurtosis and skewness into consideration, it can be argued that people tend to support the government in establishing unemployment subsidies more consistently in 2021 than in 2020. However, it is also evident in Figure 1 that after 1 year, among the respondents of the top socio-economic class, the proportion of support for unemployment subsidies has increased. In contrast, the proportion has decreased for their bottom class counterparts. This finding is interesting, so the authors constructed three sets of HGLMs to analyze further whether urban Chinese residents’ attitudes toward unemployment benefits converge or diverge under the influence of COVID-19 and to identify which factors have a significant impact on them.

Table 2 presents four HGLMs constructed from 2020 data, wherein brief, both individual and situational contexts influence the level of support of urban Chinese residents regarding the government’s setting of COVID-19 unemployment subsidies. At the individual level, respondents’ current personal income significantly impacts unemployment subsidy preferences, which confirms the view of some scholars presented in the literature review. It may be because individuals with higher incomes have more pronounced opposition to redistribution, whether this redistribution is epidemic-related. At the same time, there is no significant effect on the intragenerational mobility of individuals, which the authors suggest is explained by the fact that schooling in China has changed dramatically in the last two decades. So, schooling can be somewhat biased to represent mobility within generations, especially for urban residents, whose intragenerational mobility may relate to several factors. At the same time, individuals’ understandings and beliefs about the concept of equity, which reflects their perceptions of social stratification and social inequality, have a significant negative impact on their preferences regarding unemployment benefits. Database analysis of anonymous data from private companies to track economic activity shows that high-income people significantly reduced their spending in the early stage of the COVID-19 pandemic (mid-March 2020), which led to many layoffs of low-income workers in affluent areas. In other words, the coping measures of high-income people during the pandemic may unconsciously and negatively affect the income of low-income people (Chetty et al., 2020). The difference in income sensitivity is also evidence supporting the differential attitudes of people at different economic levels toward unemployment benefits.

Data analysis of the European Social Survey (ESS) shows that national affluence significantly determines the demand for redistribution. In Europe, the richer the country is, the more likely it is to show low support for government intervention. At least in redistribution preference, the attitude split related to personal income has not disappeared but strengthened (Filetti, 2017). Similarly, many Chinese people pay more attention to the social stratification mechanism during urbanization. Also, they are more sensitive to income distribution fairness. Although many social groups benefit from fast economic development, persistent income inequality poses a significant threat to the people in the current middle class. While income inequality lessens the earnings of the middle class, those in the low socio-economic classes, such as peasants and workers, hold a hostile attitude toward the middle class. However, the middle class earns income through legal approaches. Therefore, an invisible ideological gap emerges between the middle and bottom classes with increased income inequality in contemporary China. Consequently, the ideological gap between the middle and bottom classes can cultivate a populist ideology, especially in a period of economic transition.

Most variables significantly impact respondents’ preferences for unemployment benefits at individual and situational levels in both surveys, implying that urban Chinese residents’ perceptions of the redistribution of wealth and income represented by unemployment benefits will adjust to different situations. Optimists are less likely to support COVID-19 unemployment benefits based on past experiences or expectations of the future, and similar results show for social mobility—respondents’ perceptions of fairness influence their views on COVID-19 unemployment benefits and redistribution. Unemployment benefits are negative if people attribute personal success to effort or if poverty is perceived as an unwillingness to work.

In the HGLM of Table 4, which combines the 2020 and 2021 datasets, schooling, income, and age are estimated to be significant predictors of people’s demand for redistribution. The first two negatively correlate with the need for redistributive policies. Since educational attainment and income are both measures of people’s socio-economic status, these two negative associations confirm the adequacy of the theoretical perspective of self-interest. Low-income people have high support for the redistribution policy, and the evidence shows that when the distribution impact of the policy is clear, citizens can transform their interests and general attitudes into consistent redistribution preferences (Franko et al., 2013). Studies have shown that politically unsophisticated citizens tend to consider their interests in their attitude toward tax policy. People’s attention to distributive justice stems from their interest motivation. Self-interest has been an essential psychological factor in tax compliance (Verboon and van Dijke, 2007; Mendoza Aviña and Blais, 2022).

Varied mechanisms of the redistribution of economic benefits over time can partly explain the positive correlation between the other factor, age, and people’s preferences for redistribution. The old generation was more subject to egalitarian and totalitarian imagery of Communist regimes, especially in the Mao era, which sharply contrasts with income inequality over the last few decades. On the other hand, the young generation was born and brought up. With the dilution of abolishing the egalitarian principle, the younger generation has become more salient to accept income inequality. Accordingly, their preferences for redistribution to overcome the income gap between the rich and the poor seem to be lower than their parents’ (Zhou, 2004). Moreover, since the edu* wave and income* wave values are both positive, the explanatory power of schooling and income in people’s demand for redistribution has been suggested to be greater in 2021 than in 2020. This point again confirms that self-interest is an insightful perspective to explain whether people are in favor of or against the government’s redistributive policies.

The Chinese government is also actively exploring employment stabilization policies in response to the COVID-19 pandemic and evaluating the feasibility of these policies in practice. However, Zhang (2020) indicates that the government should formulate employment stabilization policy measures introduced during the COVID-19 pandemic under an initial policy framework designed only for employees with a clear employment relationship, which is incompatible with the current employment structure Mode does not match. Moreover, access to employment stabilization policy measures is limited because some of the most affected groups of workers do not get covered by the policy. Given the problems and difficulties encountered in implementing these policies, the future direction of employment security and social insurance reforms needs to adapt to the changing employment structure and patterns in Chinese cities.

The COVID-19 pandemic has had a more significant impact on the employment of Chinese migrant workers than urban residents. According to Che et al. (2020), as of the end of February 2020, more than 90% of rural *Hukou* (household registration) workers could not find a job, compared with 42% of *Hukou* (household registration) workers. It is difficult for the government’s unemployment assistance policies to cover migrant workers, and the enormous mobile population in cities is also facing a similar situation. COVID-19 has exacerbated inequalities arising from differences in household registration status. More substantive reforms to unemployment benefits policies thus allow China’s rural population, especially migrant workers who come to work in cities, to integrate into the national social safety net and need protection in any crisis.

COVID-19 has had a massive impact on almost all social classes worldwide, and in this context, many countries have adopted corresponding unemployment benefits (Mitman and Rabinovich, 2021). Osiander et al. (2021) explore which unemployment benefits are equitable for different groups in Germany during the COVID-19 pandemic. Find that extending unemployment insurance periods provides stable benefits to people’s income status and can provide time to find a suitable job, thereby improving the quality of the match. The attitudes of Chinese urban residents toward establishing unemployment benefits are similar to those found in Germany, but there is a more discrete consensus. The COVID-19 pandemic has forced millions to stay at home and left many unemployed in Spain. Increased appliance uses and low incomes make energy poverty more likely. Bienvenido-Huertas (2021) finds that unemployment assistance can help alleviate energy poverty, especially for those unemployed in low-paying jobs or working only a few hours a week. Kei-Ichiro and Tomoki (2022) show that Japan’s reduced working hours and increased coronavirus-related paid leave during the COVID-19 pandemic have resulted in a much lower unemployment rate than other G7 countries. The above policy measures may also be worth learning by the Chinese government. Through the government’s precise financial project approval, the economic income level of workers will not be significantly impacted by COVID-19.

## Limitations and Conclusion

The existing literature on people’s preferences for redistribution has dementedly focused on individual-level predictors rather than situational-level factors. Undoubtedly, the latter is also of great importance. Social stratification theory is significant in the real world during COVID-19, especially concerning the income implications for different groups in China. The authors combined the existing literature to divide Chinese urban residents into five socio-economic levels and analyze the variation in their attitudes toward income redistribution in the form of subsidies while also considering the impact of income inequality and regional economic development gaps. There are several intriguing findings as follows. First, consistent with the existing literature, individual-level factors (i.e., self-interest and fairness belief) significantly affect Chinese urban residents’ demand for income redistribution and government support during COVID-19. Second, the marketization index describes the socio-economic context in which respondents live and behave. We find that Chinese urban residents are more likely to oppose government provision of unemployment subsidies if they are in an environment with a higher level of marketization. Third, respondents in different socio-economic classes have different attitudes toward unemployment subsidies, and their attitudes are related to the degree of income inequality. Compared to other groups, people from the bottom level are sensitive to income inequality, and most of them explicitly support the government provision of unemployment subsidies. Respondents in the middle level are relatively moderate, while those in the higher socio-economic class are less likely to support COVID-19 unemployment subsidies. This phenomenon reflects how different groups were affected during the epidemic and how they expressed the need for income redistribution.

Having seen the sharp variances of different economic class levels in the Chinese government policy on wealth redistribution, we face the following question: is Chinese society becoming polarized? Based on our results, we must admit that we are not sure enough to give a yes or no answer. However, unemployment subsidies can enhance social resilience and help reduce inequality through income redistribution. The variances in the attitudes of urban residents of different socio-economic classes toward unemployment subsidies reflect the different expectations of the public regarding economic growth and their own lives. The CCP’s development goal of shared prosperity is a necessary correction to this trend. In the future, even after the epidemic is over, China should still focus attention on and adhere to reducing social inequality and avoiding greater social polarization.

We should address that this article has some limitations. First, subject to the impact of research time and epidemic situation, the research develops from a survey of urban residents. They do not involve rural residents, a large population in China. Therefore, the authors cannot extend the conclusion of this study to rural residents without in-depth investigation and cannot reflect the attitude of all citizens. Even for the attitude toward the unemployment compensation policy, rural residents are likely to show a completely different attitude from urban residents. We know that the Chinese government has long introduced preferential policies for the income of rural residents, such as the targeted poverty alleviation policy and the Rural Revitalization policy implemented after the Chinese government announced the complete elimination of poverty in 2021.

These policies have alleviated the severity of economic class differentiation in rural areas. Their attitude toward the unemployment subsidy policy during this epidemic is likely to be different from that in cities. Second, residents’ attitudes toward establishing unemployment benefits through taxation may be more complex, which needs some in-depth interview cases to explore. However, it is regrettable that this study does not involve the use of qualitative cases for in-depth analysis, and this will also be a topic for the authors to study in-depth in the future.

## Data Availability Statement

The original contributions presented in the study are included in the article, further inquiries can be directed to the corresponding author.

## Ethics Statement

Ethical review and approval was not required for the study on human participants in accordance with the local legislation and institutional requirements. Written informed consent from the participants was not required to participate in this study in accordance with the national legislation and the institutional requirements.

## Author Contributions

YZ played a significant role in the data analyses. JZ wrote the manuscript. YL contributed importantly to the analysis with constructive discussions. DR put forward some suggestions for improvement which helped to revise the manuscript. All authors contributed to the article and approved the submitted version.

## Conflict of Interest

The authors declare that the research was conducted in the absence of any commercial or financial relationships that could be construed as a potential conflict of interest.

## Publisher’s Note

All claims expressed in this article are solely those of the authors and do not necessarily represent those of their affiliated organizations, or those of the publisher, the editors and the reviewers. Any product that may be evaluated in this article, or claim that may be made by its manufacturer, is not guaranteed or endorsed by the publisher.

## References

[B1] Bienvenido-Huertas D. (2021). Do unemployment benefits and economic aids to pay electricity bills remove the energy poverty risk of Spanish family units during lockdown? A study of COVID-19-induced lockdown. *Energy Policy* 150 112–117. 10.1016/j.enpol.2020.112117 PMC9759601 36568910

[B2] Che L. Du H. Chan K. W. (2020). Unequal pain: a sketch of the impact of the Covid-19 pandemic on migrants’ employment in China. *Euras. Geograp. Econom.* 61 448–463.

[B3] Chetty R. Friedman J. Hendren N. (2020). *How Did COVID-19 and Stabilization Policies Affect Spending and Employment? A New Real-Time Economic Tracker Based on Private Sector Data.* Cambridge, MA: National Bureau of Economic Research, 10.3386/w27431

[B4] China Ministry of Education (2019). *In 2020 the College Graduates Will Reach 8.74 Million.* Available online at: http://www.moe.gov.cn/jyb_xwfb/s5147/201911/t20191101_406366.html. (accessed January 20, 2022).

[B5] China National Bureau of Statistics (2020). *Zhang Yi: Unemployment Rises Amid Epidemic Shock.* Available online at: http://www.stats.gov.cn/xxgk/jd/sjjd2020/202003/t20200316_1764909.html (accessed September 10, 2021).

[B6] Di Maggio P. Evans J. Bryson B. (1996). Have American’s social attitudes become more polarized? *Am. J. Sociol.* 102 690–755. 10.1371/journal.pone.0075637 24098391 PMC3788812

[B7] Filetti A. (2017). The decline of self-interest: reality or myth? analyzing the polarisation of opinions across European societies. *Eur. Politic. Sci.* 16 60–78. 10.1057/eps.2015.95

[B8] Franko W. Tolbert C. J. Witko C. (2013). Inequality, self-interest, and public support for “Robin Hood” tax policies. *Politic. Res. Quart.* 66 923–937. 10.1177/1065912913485441

[B9] Gao W. (2020). The impact of COVID-19 on employment in China and its response. *J. Graduate School Chin. Acad.* 03 21–31.

[B10] Jaime-Castillo A. M. Sáez-Lozano J. L. (2016). Preferences for tax schemes in OECD countries, self-interest and ideology. *Int. Politic. Sci. Rev.* 37 81–98. 10.1177/0192512114539716

[B11] Kei-Ichiro I. Tomoki M. (2022). Japan’s unemployment rate hike amid the COVID-19 pandemic - why was it so mild? *Appl. Econom. Lett.* 2022:2031857. 10.1080/13504851.2022.2031857

[B12] Kim Y. J. Cho J. H. Kim E. S. (2020). Differences in sense of belonging, pride, and mental health in the Daegu metropolitan region due to COVID-19: comparison between the presence and absence of national disaster relief fund. *Int. J. Environ. Res. Public Health* 17:4910. 10.3390/ijerph17134910 32646034 PMC7370047

[B13] Li C. (2020). Employment of college students under the impact of the COVID-19: employment pressure, psychological pressure, and changes in employment choices. *Educat. Res.* 47 21–31.

[B14] Li Y. (2020). The urban-rural differences in redistribution preference: an empirical study based on social status and fairness. *China Economic Stud.* 2020 92–105. 10.19365/j.issn1000-4181.2020.01.07

[B15] Li Y. Lv G. (2019). Perception of income fairness, prospect of mobility and preference for redistribution: empirical evidence from CGSS 2013. *Finance Trade Econom.* 40 35–49.

[B16] Mendoza Aviña M. Blais A. (2022). Are tax cuts supporters self-interested and/or partisan? the case of the tax cuts and jobs act. *Am. Polit. Res.* 50, 416–427. 10.1177/1532673x211041147

[B17] Mitman K. Rabinovich S. (2021). Whether, when and how to extend unemployment benefits: theory and application to COVID-19. *J. Public Economics* 200:104447. 10.1016/j.jpubeco.2021.104447 34934254 PMC8677353

[B18] Moon J. Y. Sun Y. M. (2021). The effect of COVID-19 emergency disaster relief fund on welfare attitude. *Kor. J. Soc. Welfare* 73 29–55.

[B19] Nemteanu M.-S. Dinu V. Dabija D.-C. (2021). Job insecurity, job instability, and job satisfaction in the context of the COVID-19 pandemic. *J. Competitiv*. 13 65–82. 10.7441/joc.2021.02.04

[B20] Newman B. J. Teten P. (2021). Inequality federalism and economic self-interest in subnational progressive tax politics. *Politic. Res. Quart.* 74 243–252. 10.1177/1065912920905111

[B21] Olssen M. (2021). The rehabilitation of the concept of public good: reappraising the attacks from liberalism and neoliberalism from a poststructuralist perspective. *Rev. Contemp. Philos.* 20 7–52. 10.22381/rcp2020211

[B22] Osiander C. Senghaas M. Stephan G. Struck O. (2021). Längeres arbeitslosengeld in der krise? Covid-19 und die angemessene maximale Bezugsdauer. *Kolner Zeitschrift Fur Soziologie Sozialpsychol*. 73 419–448. 10.1007/s11577-021-00806-3 34898722 PMC8647966

[B23] Rin K. S. (2021). Comparison of youtube comments on multicultural citizens of Korea and Japan over COVID-19 emergency relief funds. *J. Kor. Contents Associat.* 21 112–120.

[B24] Sainz M. Martínez R. Rodríguez-Bailón R. Moya M. (2019). Where does the money come from? humanizing high socio-economic status groups undermines attitudes toward redistribution. *Front. Psychol.* 10:771. 10.3389/fpsyg.2019.00771 30984094 PMC6450225

[B25] Shen Y. Li S. Wang X. (2021). Impacts of two tax reforms on inequality and welfare in China. *China World Economy* 29 104–134. 10.1111/cwe.12377

[B26] Tinghög G. Andersson D. Västfjäll D. (2017). Are individuals luck egalitarians? - an experiment on the influence of brute and option luck on social preferences. *Front Psychol.* 8:460. 10.3389/fpsyg.2017.00460 28424641 PMC5372824

[B27] Verboon P. van Dijke M. (2007). A self-interest analysis of justice and tax compliance: how distributive justice moderates the effect of outcome favorability. *J. Economic Psychol.* 28 704–727. 10.1016/j.joep.2007.09.004

[B28] Walker H. M. Lev J. (1969). *Elementary Statistical Methods*, 3rd Edn. New York, NY: Holt, Rinehart & Winston, 59–69.

[B29] Wu X. Zhou Y. (2020). Thoughts on ‘replacing taxation from premiums’ in social insurance: based on the employment situation under the influence of COVID-19. *Taxation Res.* 4 37–40. 10.19376/j.cnki.cn11-1011/f.2020.06.007

[B30] Xiao Y. (2021). Sense of social fairness, redistribution preference and welfare attitude: an empirical analysis based on CGSS 2015 data. *J. Dalian Univ. Technol.* 42 101–109. 10.19525/j.issn1008-407x.2021.03.012

[B31] Xu J. Liu H. (2013). Social justice recognition, fluidity expectation and preference of residence redistribution– an empirical study based on CGSS data. *J. Yunnan Univ. Finan. Econom.* 29 48–56. 10.16537/j.cnki.jynufe.2013.02.011

[B32] Xu Z. (2018). The populist tendency in collective action and its regulation. *Acad. Forum.* 41 141–147. 10.16524/j.45-1002.2018.05.018

[B33] Zhan P. Li S. Xu X. (2019). Personal income tax reform in China in 2018 and Its impact on income distribution. *China World Economy* 27 25–48. 10.1111/cwe.12279

[B34] Zhang G. Wu T. (2020). Research on the impact of the COVID-19 on employment in China. *Chin. Populat. Sci.* 11 11–20.

[B35] Zhang H. (2020). China’s employment stabilization policies in response to the impact of the COVID-19 pandemic. *Int. J. Sociol. Soc. Policy* [Online ahead-of-print]. 10.1108/ijssp-05-2020-0167

[B36] Zhou X. (2004). *The State and Life Chances in Urban China: Redistribution and Stratification, 1949–1994.* Cambridge: Cambridge University Press. 10.1017/CBO9780511499401

