# Supplementary material for: Convergence or Divergence: Preferences for Establishing an Unemployment Subsidy During the COVID-19 Period by Taxing Across Earnings Redistribution in Urban China
Source: Front Psychol. 2022 Jun 10;13:852792. doi: 10.3389/fpsyg.2022.852792 (PMC9226365; doi:10.3389/fpsyg.2022.852792)
Supplement: Supplementary file 1 [file Data_Sheet_1.docx]

**Appendix:** **distribution of data**

***Table. Variables in 2020***

| **variable** | **Number/Mean** | |
| --- | --- | --- |
|  | **Percentage** | **Mean** |
| **Economic class** |  |  |
| top | 6% |  |
| Middle-top | 25% |  |
| Middle | 37% |  |
| Middle-bottom | 29% |  |
| Bottom | 3% |  |
| ***Control variable*** |  |  |
| Age |  | 42 |
| Gender(reference=male) | 51% |  |
| Employment status (reference=employed) | 79% |  |
| Marital status (reference=married) | 92% |  |
| Schooling |  | 15.7 |
| Welfare |  | 2.8 |
| ***Individual-level*** |  |  |
| **Self interest** |  |  |
| Current personal income(log) |  | 5.29 |
| Past comparison income (reference=bad) | 32% |  |
| *better* | 27% |  |
| *same* | 41% |  |
| Current comparison income (reference=bad) | 23% |  |
| *better* | 56% |  |
| *same* | 21% |  |
| Intragenerational mobility (edu.) |  | 1.67 |
| **Fairness belief** |  |  |
| Existence of the rich and the poor (reference=disagree) | 42% |  |
| Children’s equal access to education (reference=disagree) | 78% |  |
| Equal opportunities of descents of workers or peasants to become high-socioeconomic-status people (reference=disagree) | 85% |  |
| ***Situational-level*** |  |  |
| Marketization index | 6.73 |  |
| Gini Coefficient | 0.37 |  |
| ***N*** | 4694 |  |

***Table. Variables in 2021***

|  |  | |
| --- | --- | --- |
|  | **Percentage** | **Mean** |
| **Economic class** |  |  |
| top | 5% |  |
| Middle-top | 26% |  |
| Middle | 38% |  |
| Middle-bottom | 24% |  |
| Bottom | 7% |  |
| ***Control variable*** |  |  |
| Age |  | 43.2 |
| Gender(reference=female) | 51.6% |  |
| Employment(reference=unemployed) | 85% |  |
| Married(reference=unmarried) | 95% |  |
| Education |  | 15.3 |
| Welfare |  | 2.76 |
| ***Individual-level*** |  |  |
| **Self interest** |  |  |
| Current personal income(log) | 5.87 |  |
| Past comparison income (reference=bad) | 45% |  |
| *better* | 15% |  |
| *same* | 40% |  |
| Past promotion experience (reference=Yes) | 65% |  |
| Past wage increase experience (reference=Yes) | 61% |  |
| Future promotion expectation (reference=Yes) | 25% |  |
| Future wage increase expectation (reference=Yes) | 32% |  |
| Intragenerational mobility (occupation) |  | 1.7 |
| Intragenerational mobility (edu.) |  | 2.4 |
| **Fairness belief** |  |  |
| Success due to luck | 46% |  |
| Success due to social network | 79% |  |
| Success due to individual capacity | 83% |  |
| Being poor due to idleness (reference=disagree) | 39% |  |
| Being poor due to education insufficiency (reference=disagree) | 59% |  |
| Being poor due to government’s policy (reference=disagree) | 19% |  |
| ***Situational-level*** |  |  |
| Marketization index | 6.95 |  |
| Gini Coefficient | 0.39 |  |
| ***N*** | 5205 |  |
